# AI literacy and high school English teachers' teaching anxiety: the chain mediation role of teaching self-efficacy and work engagement

**DOI:** 10.3389/fpsyg.2026.1797047

**Published:** 2026-07-08

**Authors:** Shile Cao, Xiaoli Liu, Xiaohang Yu

**Affiliations:** 1 Southwest University, Chongqing, China; 2 Department of General Education, Qilu Medical University, Zibo, China

**Keywords:** AI literacy, EFL teachers, serial mediation, teaching anxiety, teaching self-efficacy, work engagement

## Abstract

**Introduction:**

This study explores the impact of AI literacy on high school EFL teachers' teaching anxiety, with a particular focus on the underlying psychological mechanisms. As AI technologies become increasingly embedded in educational contexts, teachers are expected to adapt to new digital tools. While AI literacy may offer practical benefits, its relationship with teaching anxiety through psychological and motivational pathways remains underexplored. This study aims to fill this gap by examining the serial mediation roles of teaching self-efficacy and work engagement in the relationship between AI literacy and teaching anxiety.

**Methods:**

A quantitative research design was employed to examine the hypothesized relationships. Data were collected from a sample of 392 high school English teachers in China using 5-point Likert scales. The responses were analyzed using SmartPLS to conduct partial least squares structural equation modeling (PLS-SEM). The analysis included assessments of measurement model reliability and validity, followed by structural model evaluation. Mediation effects were tested through bootstrapping, and the Variance Accounted For (VAF) was calculated to assess the strength of indirect effects. While most serial mediation studies use SPSS with PROCESS, this study employed PLS-SEM, a relatively novel approach in educational research, to explore chain mediation.

**Results:**

The results revealed that AI literacy negatively affected teaching anxiety, with higher AI literacy linked to lower anxiety. Both teaching self-efficacy and work engagement mediated this relationship, with self-efficacy serving as a stronger mediator. A significant serial mediation pathway was found: AI literacy was associated with higher self-efficacy, which in turn was related to increased work engagement and lower teaching anxiety.

**Discussions:**

The results provide support for the proposed hypotheses and highlight the significant role of AI literacy in relation to psychological well being among EFL teachers. The findings suggest that AI literacy is associated with higher self-efficacy and engagement, which are in turn linked to lower teaching-related anxiety. These findings point to the importance of integrating AI literacy training alongside psychological and motivational support strategies in teacher development programs, particularly within the evolving landscape of AI-driven education.

## 1 Introduction

Teaching anxiety refers to the tension and stress that teachers experience when confronting challenges in their teaching practice, such as heavy lesson preparation, classroom management difficulties, time constraints, and pressure to meet curriculum standards (Aydin, 2021; Henderson and Corry, 2021). For English as a Foreign Language (EFL) teachers, this anxiety can be more pronounced due to the complexity involved in teaching various language skills, including speaking, writing, listening, and reading (Irhamna and Fithriani, 2023).

The negative impact of teaching anxiety on EFL teachers is substantial and should not be overlooked. According to Aydin (2021), high levels of teaching anxiety can undermine teachers' confidence, motivation, and self-esteem, ultimately impairing their classroom performance. Agyapong et al. (2022) further noted that teaching anxiety may contribute to teacher burnout, decreased job satisfaction, and an increased risk of resignation. Prolonged teaching anxiety can also result in psychological health issues such as depression and manifest in physical symptoms like sleep disturbances (Biernat et al., 2022). Moreover, teachers experiencing high anxiety levels may unintentionally transmit this anxiety to students, negatively affecting students' motivation and academic performance (Yue et al., 2023). Therefore, it is essential to implement effective strategies to reduce teaching anxiety among EFL teachers.

In recent years, Artificial Intelligence (AI) has been widely applied across various educational contexts, initiating a significant transformation in the field of education (Chen et al., 2022). AI literacy refers to the ability to understand and critically evaluate AI systems, effectively communicate and collaborate with AI, and utilize AI tools in online, home, and workplace settings (Du et al., 2024). Compared to their counterparts with lower AI literacy, EFL teachers with higher levels of AI literacy are more capable of leveraging AI to generate lesson plans and educational resources, thereby saving time and alleviating the pressure of lesson preparation (Koraishi, 2023). Additionally, they can utilize AI to personalize learning materials based on students' individual learning styles, pace, and needs, which enhances teaching effectiveness (Zhang and Zhang, 2024).

Teachers with advanced AI literacy are also more likely to integrate AI into their daily teaching practices, employing AI to support student learning and efficiently assess academic performance. AI tools can analyze students' performance patterns, enabling teachers to identify those who need additional support and adjust their instructional strategies accordingly (Mohamed, 2024). Moreover, AI-literate EFL teachers are better equipped to explore and implement innovative teaching methods and tools, making their instruction more engaging and impactful (Williyan et al., 2024). Therefore, enhancing EFL teachers' AI literacy not only helps them adapt to emerging technologies and educational trends but also contributes to their professional development and long-term career advancement.

Currently, there is a relative paucity of research examining the impact and underlying mechanisms of AI literacy on teaching anxiety among EFL teachers. To address this gap, the present study investigates how EFL teachers' AI literacy influences their teaching anxiety. By introducing teaching self-efficacy and work engagement as mediating variables, this study constructs a chain mediation model to gain a deeper understanding of the pathways through which AI literacy affects teaching anxiety.

## 2 Literature review

### 2.1 AI literacy and teaching anxiety

Teachers' teaching anxiety is influenced by various factors. Zhukova (2018) argued that novice teachers often experience higher levels of teaching anxiety due to their lack of teaching experience. Henderson and Corry (2021) pointed out that a lack of professional expertise in the subjects they teach can prevent teachers from adopting effective instructional strategies, resulting in poor teaching performance and heightened anxiety. In addition, the heavy workload associated with teaching such as course design and student assessments may overwhelm teachers and contribute to increased teaching anxiety (Heffernan et al., 2022).

With the rapid development of artificial intelligence (AI), its application in education is becoming increasingly widespread. AI can assist teachers in various aspects of the teaching process. For example, by analyzing educational trends and existing gaps, AI can help teachers design instructional plans that ensure the relevance and consistency of teaching content with curriculum standards (Jiménez, 2024). Certain AI systems, such as EduAide, are capable of developing personalized teaching plans tailored to students' unique needs (Georgia et al., 2024). Moreover, AI can assess students' performance in real time and generate customized practice materials based on the evaluation data, allowing instruction to adapt to students' individual learning progress. This significantly reduces teachers' workload in classroom teaching (Chen et al., 2020). In addition, AI can create engaging educational games that enhance student motivation and alleviate teachers' classroom management pressure (Benzizoune, 2024).

However, the trend of integrating AI technology into education has also intensified teaching anxiety among some teachers. Alam (2021) pointed out that, due to the powerful automation capabilities of AI, it can easily perform tasks that were traditionally carried out by educators. As a result, some teachers fear that AI may eventually replace their roles, diminishing their significance in the teaching process or even leading to unemployment. Moreover, with the rapid advancement of AI technology, an increasing number of school administrators are requiring teachers to incorporate AI into their teaching. Teachers who lack sufficient understanding of AI and have limited ability to use AI tools may experience anxiety due to their inability to effectively integrate AI into their instructional practices (Melweth et al., 2024). Additionally, some teachers believe that the use of AI in education might negatively affect students, which makes them feel anxious and concerned about this new mode of teaching (Felix, 2020). A key reason behind these concerns is the low level of AI literacy among teachers, including their limited understanding of AI and insufficient skills to apply it effectively.

Zhang and Zhang (2024) stated that providing AI training and enhancing teachers' AI literacy can help them better understand both the advantages and limitations of AI technology, and become more proficient in its use. This enables teachers to make reasonable and effective use of AI to support their teaching, thereby reducing their teaching anxiety. Teachers with a high level of AI literacy are able to delegate non-instructional tasks to AI, allowing them to concentrate more on core teaching activities. This can help alleviate the pressure and anxiety caused by heavy workloads (Ding et al., 2024). Furthermore, teachers with high AI literacy tend to have a more objective and rational understanding of the impact of AI on classroom teaching. They are more likely to view AI as a supportive tool rather than a threat, and thus experience lower levels of anxiety regarding the integration of AI into their teaching practices (Kim, 2024).

### 2.2 The mediating role of teaching self-efficacy

Teaching self-efficacy refers to the belief of teachers that they can effectively handle tasks and challenges related to their teaching, including successfully completing teaching content design, organizing classroom activities reasonably, and promoting students' academic development (Barni et al., 2019; Lazarides and Warner, 2020). Studies have found that teachers' AI literacy is positively associated with their teaching self-efficacy. Seunmin and Anna (2024) conducted a study on the relationship between Al literacy, teaching self-efficacy, and professional identity among Korean EFL teachers. The results showed that teachers' AI literacy was significantly and positively associated with their teaching self-efficacy and professional identity. Teachers with higher AI literacy often exhibit a more positive and confident teaching attitude, and they are more likely to view AI as a tool to enhance their teaching effectiveness. Zainal and Mohd Matore (2024) pointed out that teachers with high AI literacy are more proficient in using AI tools in teaching. They are better at using AI to assist themselves in completing teaching tasks smoothly. This greatly enhances teachers' confidence in their teaching abilities and makes them have a more positive attitude toward teaching. Therefore, enhancing teachers' AI literacy can help them overcome concerns about teaching and build confidence. As teachers become more familiar with AI tools, they will use them more effectively in their teaching, which can enhance their teaching self-efficacy (Hur, 2025).

This study draws on two complementary theoretical frameworks. Social cognitive theory is employed to explain the mediating role of teaching self-efficacy, as social cognitive theory directly links perceived competence to emotional outcomes. Self-determination theory is used to explain the mediating role of work engagement, as Self-determination theory focuses on intrinsic motivation and psychological need satisfaction. According to social cognitive theory, self-efficacy is a key factor determining individual behavior and performance. People with high self-efficacy are more likely to cope with challenges with confidence and less anxiety (Li, 2023). Teachers with high self-efficacy tend to believe in their ability to effectively manage classrooms, plan courses, and achieve teaching goals, and they are less likely to experience teaching anxiety (Lazarides and Warner, 2020). Some studies have also reported a negative association between teachers' teaching self-efficacy and teaching anxiety. Chen Musgrove and Schussler (2020) conducted a study on the factors influencing teaching anxiety among biology graduate assistants in the United States. The research results found that higher teaching self-efficacy is associated with lower teaching anxiety, indicating a negative association between the two variables. Asare (2025) conducted a study on the relationship between teaching self-efficacy, teaching enthusiasm, and teaching anxiety among pre-service management teachers. The research results indicated a negative correlation between the teaching self-efficacy of pre-service management teachers and their teaching anxiety. Xiyun et al. (2022) stated that higher teachers' teaching self-efficacy is associated with greater confidence in dealing with challenging teaching situations and lower teaching anxiety. Therefore, teachers' AI literacy may be associated with their ability to use AI reasonably in teaching, and may be related to higher confidence in English teaching and lower levels of teaching anxiety.

### 2.3 The mediating role of work engagement

Many researchers considered that enhancing teachers' AI literacy can promote their work engagement. Du et al. (2024) pointed out that teachers with higher level of AI literacy can use AI tools more effectively, thereby improving their teaching abilities and teaching performance. This will increase teachers' satisfaction and dedication to their work, as they have higher confidence and identification with their teaching role. Chiu and Rospigliosi (2025) argued that if teachers have high AI usage abilities, they can use AI to help themselves in their work, greatly reducing their workload. The reduction of workload can alleviate teachers' work pressure and enable them to be more focused and engaged in their work. Al-Abdullatif (2024) stated that teachers with high AI literacy are better equipped to adapt to the educational changes brought about by AI technology and adopt new teaching methods in their own teaching. This adaptability helps to increase teachers' work engagement, as they are more likely to accept new challenges and opportunities.

According to self-determination theory, when teachers' intrinsic motivation and engagement in teaching increase, their ability to manage stress is stronger, and therefore negative emotions toward teaching will also decrease (Uysal, 2023). Studies have shown that teachers' work engagement is negatively associated with their teaching anxiety. Hakanen et al. (2006) conducted a questionnaire survey of 2045 Finnish teachers and found that a supportive work environment can enhance teachers' work engagement and reduce their occupational anxiety. Tao (2022) conducted a study on the relationship between mindfulness, work engagement, and classroom emotions among Chinese primary and secondary school teachers. The research results found that teachers' mindfulness is positively correlated with their work engagement, and the increase in teachers' work engagement can reduce their negative emotions such as anxiety in the classroom. Fute et al. (2022) found that the work engagement of teachers is negatively correlated with their negative emotions (fatigue, anxiety, etc.) in teaching. The work engagement of teachers is crucial in reducing their negative emotions and maintaining job satisfaction. Therefore, teachers' AI literacy may be associated with their enthusiasm to explore new technologies and teaching methods, work engagement in English teaching, and teaching anxiety.

### 2.4 The chain mediating role of teaching self-efficacy and work engagement

Studies have shown a positive correlation between teachers' teaching self-efficacy and their work engagement (Han and Wang, 2021; Heng and Chu, 2023). Xiao et al. (2022) noted that teachers with higher self-efficacy in teaching are more confident in their ability to manage the classroom, organize lessons, and achieve teaching goals, which enhances their enthusiasm for teaching activities and boosts their work engagement.

According to self-determination theory, teachers' AI literacy may be associated with their teaching self-efficacy, potentially through the satisfaction of basic psychological needs. As teachers' self-efficacy improves, their work engagement also increases. The ability to effectively use AI enables teachers to apply AI-assisted teaching in ways that align with their needs. This autonomy allows them to make informed decisions on utilizing AI tools, ensuring that their teaching strategies are in harmony with their goals and values, which further strengthens their self-efficacy and increases work engagement (Xia et al., 2022). Improving teachers' AI literacy also enhances their ability to manage complex educational technologies. This capability leads to better classroom management techniques and more effective teaching strategies, which in turn further boosts their teaching self-efficacy. As teachers' confidence in their abilities grows, they are more likely to fully invest in their work (Zhang et al., 2021).

In addition, when teachers possess strong skills in using AI, they are better equipped to collaborate with colleagues who share similar interests in AI-assisted teaching. This collaboration can lead to mutual support and professional growth, while also increasing recognition from school administrators regarding their teaching competencies. Such a sense of collaboration and belonging may be associated with a supportive work environment in which teachers feel valued and acknowledged. This, in turn, is associated with higher levels of teaching self-efficacy and work engagement (Bergdahl et al., 2023; Tafvelin and Stenling, 2021). Therefore, teachers' AI literacy may be associated with teaching self-efficacy, which may in turn be related to work engagement and teaching anxiety.

### 2.5 The current study

**Figure 1:**
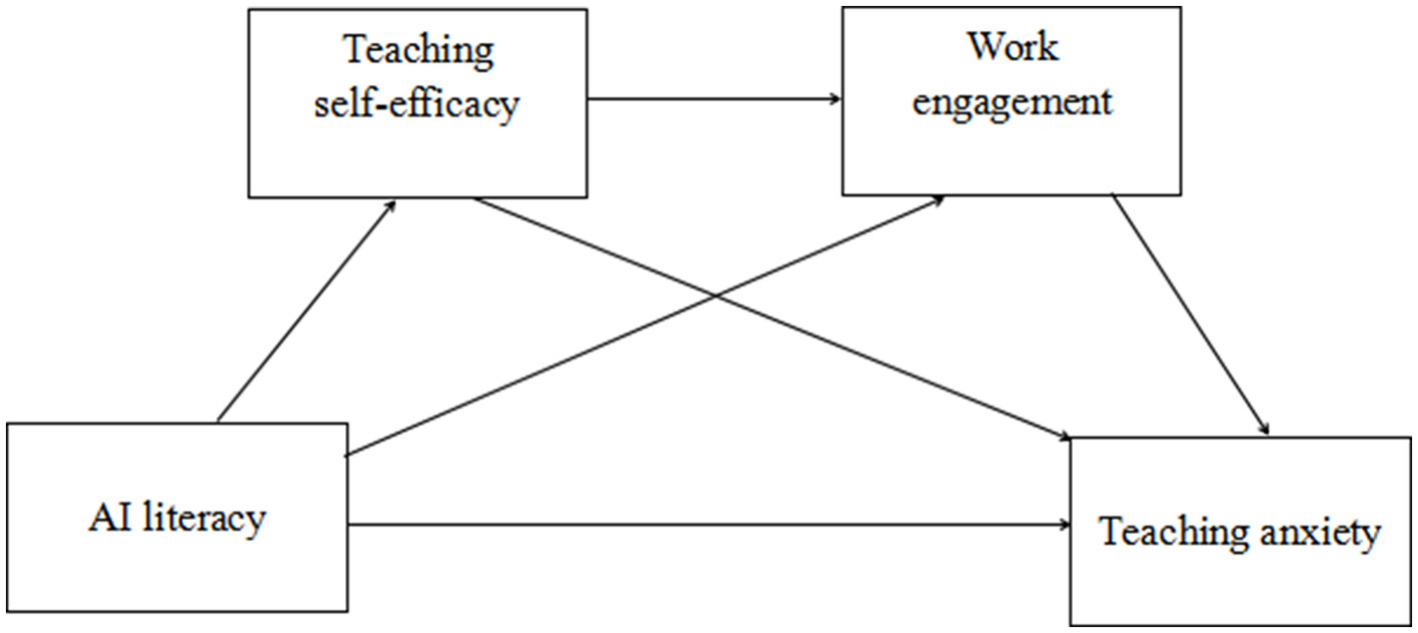
Hypothesized research model of this study.

In this study, we constructed a chain mediation model based on a review of existing literature and proposed hypotheses to explore the influence of EFL teachers' AI literacy on their teaching anxiety, with teaching self-efficacy and work engagement serving as mediators (as shown in Figure 1).

H1: AI literacy of EFL teachers is negatively correlated with their teaching anxiety.

H2: Teaching self-efficacy mediates the relationship between AI literacy and teaching anxiety.

H3: Work engagement mediates the relationship between AI literacy and teaching anxiety.

H4: Teaching self-efficacy and then work engagement serial mediate the relationship between AI literacy and teaching anxiety.

## 3 Methodology

### 3.1 Participants

This study investigated high school English teachers from various regions across China. Participants were selected to ensure a diverse and representative sample of educators working in different educational and cultural contexts. The respondents received a questionnaire along with a cover letter assuring confidentiality. Only EFL teachers employed in high schools were eligible to participate, and participation was entirely voluntary. Data were collected *via* an online questionnaire distributed through Wenjuanxing, a widely used survey platform in China. A total of 500 questionnaires were distributed, and 405 responses were received (response rate = 81%). After screening for low-quality responses, such as identical answers (straight-lining) and extremely short completion times, 392 valid responses remained, yielding a valid response rate of 78.4%.

### 3.2 Measures

#### 3.2.1 AI literacy scale (AILS)

The AI Literacy Scale (AILS), developed by (Wang et al., 2023), is a validated instrument designed to measure individuals' competence in using artificial intelligence (AI) technologies. Based on a four-construct framework, the scale assesses AI literacy across four dimensions: (1) Awareness, referring to the ability to identify and understand AI technologies and their applications; (2) Usage, which reflects proficiency in applying AI tools to accomplish tasks; (3) Evaluation, involving critical analysis and judgment of AI outputs and applications; and (4) Ethics, which addresses the understanding of ethical responsibilities and potential risks associated with AI use. In this study, the scale uses a 5-point Likert scale ranging from 1 (Strongly Disagree) to 5 (Strongly Agree). The AILS has demonstrated good construct validity and a high level of internal consistency, with a Cronbach's alpha of 0.83.

#### 3.2.2 Ohio state teacher efficacy scale (OSTES)

The Ohio State Teacher Efficacy Scale (OSTES), developed by (Tschannen-Moran and Hoy, 2001), is an instrument used in this study to assess teachers' sense of efficacy across three core dimensions: instructional strategies, classroom management, and student engagement. Each dimension consists of four items, reflecting teachers' confidence in their ability to apply effective teaching methods, manage classroom behavior, and engage students in learning. Responses are measured on a 5-point Likert scale ranging from 1 (Strongly Disagree) to 5 (Strongly Agree). The scale has demonstrated high internal consistency in previous research, with an overall Cronbach's alpha of 0.90 and subscale alphas ranging from 0.81 to 0.86, supporting its reliability and validity for use in educational research.

#### 3.2.3 Utrecht work engagement scale (UWES)

The Utrecht Work Engagement Scale (UWES) used in this study is a self-report instrument designed to assess teachers' work engagement, which encompasses three dimensions: vigor, referring to high levels of energy and mental resilience at work; dedication, reflecting a sense of enthusiasm, pride, and meaningfulness; and absorption, characterized by being fully concentrated and deeply engrossed in work. Responses are measured on a 5-point Likert scale ranging from 1 (Strongly Disagree) to 5 (Strongly Agree). The UWES has demonstrated strong reliability and validity in prior research, including high internal consistency (α ≥ 0.85 for the total score), and is widely recognized for its applicability across diverse occupational and cultural settings.

#### 3.2.4 Teaching anxiety scale (TCHAS)

The Teaching Anxiety Scale (TCHAS), originally developed by (Parsons, 1973), is designed to measure anxiety specifically related to teaching. In this study, a revised version of the TCHAS was used to better reflect the specific context of Chinese high school English teachers. The scale adopts a 5-point Likert format ranging from 1 (Strongly Disagree) to 5 (Strongly Agree), with higher scores indicating greater levels of teaching anxiety. The TCHAS has demonstrated strong internal consistency, with reported Cronbach's alpha values ranging from 0.87 to 0.94.

### 3.3 Data analysis

After careful consideration of the study design and sample characteristics, both SPSS (Statistical Package for the Social Sciences) and SmartPLS were employed to perform the statistical analyses. SPSS was used to conduct descriptive statistics to summarize demographic characteristics and to assess common method bias through Harman's single-factor test. SmartPLS (version 4) was used as the primary tool for hypothesis testing. Partial Least Squares Structural Equation Modeling (PLS-SEM) was employed to evaluate both the measurement model (assessing indicator reliability, internal consistency, convergent validity, and discriminant validity) and the structural model (testing hypothesized relationships among constructs). To examine the mediation effects, a serial mediation analysis was conducted within the PLS-SEM framework using the bootstrapping technique with 5,000 resamples. This analysis assessed the direct effect, two specific indirect effects (*via* each mediator independently), and one serial indirect effect (through both mediators sequentially). The bootstrapped confidence intervals were used to determine the significance of the mediation pathways. This approach is particularly suitable for complex models in social science research, allowing for a nuanced understanding of how constructs interact through intermediary variables (Hair et al., 2017).

## 4 Results

### 4.1 Common method bias and variance inflation factor

Since all variables in this study were measured using self-reported data collected from high school English teachers, the possibility of common method bias (CMB) was considered. To assess the presence of CMB, the Harman single-factor test was conducted. All items from the questionnaires were entered into an exploratory factor analysis using SPSS with no rotation. The results revealed a total of four factors with eigenvalues greater than 1, indicating that the variance was distributed across multiple factors rather than being dominated by a single underlying factor. The first factor accounted for only 40.048% of the total variance (see Table 1), which is well below the commonly accepted threshold of 50%. These results suggest that common method bias is not a major concern in this study (Podsakoff et al., 2003). It should be noted that Harman's single-factor test has limitations in detecting common method bias.

**Table 1:** CMB results.

Component	Initial eigenvalues			Extraction sums of squared loadings		
	Total	% of variance	Cumulative %	Total	% of variance	Cumulative %
1	18.021	40.048	40.048	18.021	40.048	40.048
2	2.713	6.029	46.077	2.713	6.029	46.077
3	2.086	4.636	50.712	2.086	4.636	50.712
4	1.943	4.317	55.029	1.943	4.317	55.029

Since the results of the common method bias test indicated no serious bias, further analysis was conducted to examine multicollinearity using the Variance Inflation Factor (VIF). All VIF values were below the commonly accepted threshold of 5, indicating no serious multicollinearity issues (Hair et al., 2017). Details are in Table 3. Additionally, the respondents were given questionnaires and a cover letter that guaranteed confidentiality. Only EFL teachers in high schools in China were eligible to participate in this survey, and it was entirely voluntary.

### 4.2 Descriptive statistics

A total of 392 high school English teachers participated in this study. As shown in Table 2, 86% were female and 14% were male. In terms of age distribution, 28% were under 30, 26% were between 31 and 40, 38% were between 41 and 50, and 8% were above 50 years old. Regarding educational background, 14% held an Associate Degree, 40% a Bachelor's Degree, 34% a Master's Degree, and 12% a Doctoral Degree. For professional titles, 29% of the participants held junior titles, 35% intermediate titles, 27% associate senior titles, and 9% senior titles. In terms of teaching experience, 16% had 1–3 years, 17% had 4–6 years, 27% had 7–10 years, and 40% had more than 10 years of teaching experience. With regard to school type, 27% of the teachers were from urban key high schools, 35% from urban regular high schools, 26% from county/district high schools, and 12% from private high schools.

**Table 2:** Demographic profiles.

Demographic variables	Type	Frequency (*N* = 392)	Percentage (%)
Gender	Female	337	86
	Male	55	14
Age	under 30	110	28
	31–40	101	26
	41–50	149	38
	above 50	32	8
Educational level	Associate degree	53	14
	Bachelor's degree	159	40
	Master's degree	132	34
	Doctoral degree	48	12
Professional titles	Junior title	113	29
	Intermediate title	138	35
	Associate senior title	106	27
	Senior title	35	9
Years of teaching	1–3 years	64	16
	4–6 years	68	17
	7–10 years	105	27
	above 10 years	155	40
School type	Urban key high school	105	27
	Urban regular high school	140	35
	County/district high school	100	26
	Private high school	47	12

**Table 3:** Measurement model result.

Construct/items	Loadings	α	CR	AVE	VIF
*AI Literacy*		0.920	0.921	0.533	
AIL_1	0.727				1.857
AIL_2	0.707				1.731
AIL_3	0.737				1.864
AIL_4	0.758				2.027
AIL_5	0.765				2.063
AIL_6	0.706				1.745
AIL_7	0.711				1.756
AIL_8	0.707				1.749
AIL_9	0.740				1.909
AIL_10	0.738				1.913
AIL_11	0.736				1.874
AIL_12	0.723				1.796
*Teaching self-efficacy*		0.919	0.920	0.529	
TSE_1	0.739				1.924
TSE_2	0.779				2.104
TSE_3	0.711				1.807
TSE_4	0.759				2.002
TSE_5	0.707				1.779
TSE_6	0.721				1.811
TSE_7	0.715				1.818
TSE_8	0.718				1.833
TSE_9	0.701				1.733
TSE_10	0.727				1.827
TSE_11	0.718				1.769
TSE_12	0.725				1.850
*Working engagement*		0.889	0.889	0.562	
WE_1	0.744				1.799
WE_2	0.733				1.790
WE_3	0.750				1.815
WE_4	0.729				1.693
WE_5	0.749				1.807
WE_6	0.773				1.941
WE_7	0.773				1.871
WE_8	0.745				1.808
*Teaching anxiety*		0.936	0.936	0.564	
TA_1	0.765				2.175
TA_2	0.735				1.978
TA_3	0.754				1.996
TA_4	0.733				1.965
TA_5	0.771				2.140
TA_6	0.765				2.093
TA_7	0.796				2.303
TA_8	0.734				1.913
TA_9	0.730				1.911
TA_10	0.735				2.066
TA_11	0.736				1.927
TA_12	0.762				2.082
TA_13	0.745				1.973

Overall, a large proportion of the 392 high school English teachers were female, with the majority in the 41–50 age range. Most held either a Bachelor's or Master's degree and held junior or intermediate professional titles. A significant portion had more than 10 years of teaching experience. The teachers were primarily from urban regular high schools, followed by urban key, county/district, and private high schools.

**Figure 2:**
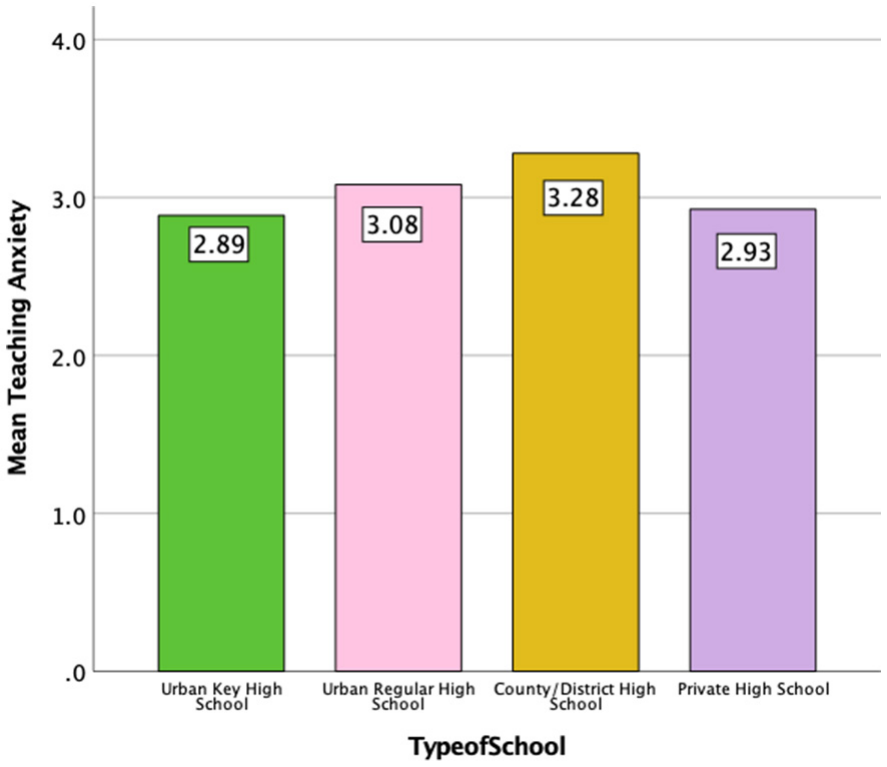
Mean of school type.

However, it is very interesting that school type has a significant difference on teaching anxiety. Figure 2 illustrates the mean levels of teaching anxiety across different types of school (urban key high school, urban regular high school, county/district high school and private high school). Teachers from county/district high schools reported the highest level of teaching anxiety (*M* = 3.17), followed by those from urban regular high schools (*M* = 2.96). Private high school teachers reported a slightly lower level of anxiety (*M* = 2.80), while the lowest level was observed among teachers from urban key high schools (*M* = 2.76). These results suggest that school context may play a role in influencing teachers' experiences of teaching anxiety.

One of these findings suggests that teachers in rural schools may experience higher levels of teaching anxiety. This is consistent with Irhamna and Fithriani (2023), who found that a lack of psychological and educational resources was a major contributing factor in rural settings. Novice teachers, in particular, often feel anxious due to insufficient knowledge to manage unexpected questions from students, leading to nervousness in classroom situations. Muremela et al. (2021) further supported this by highlighting that the quality of teaching in rural areas is frequently hindered by limited support and resources. Similarly, DiRliK et al. (2021) acknowledged that place of residence can influence anxiety levels, suggesting that urban environments, due to their greater access to educational and cultural resources, may be associated with lower levels of anxiety among teachers compared to their rural counterparts.

Another notable finding is that teachers in ordinary urban high schools tend to report higher levels of teaching anxiety compared to those in key high schools, which is supported by past studies. Research suggests that key high schools offer a more stable and supportive teaching environment, with clearer expectations and more academically prepared students, which reduces stress and behavioral challenges (González et al., 2023; Ratanasiripong et al., 2020). In contrast, teachers in ordinary schools may face greater pressure due to varied student performance and classroom management demands. During the COVID-19 pandemic, teachers in key high schools also reported lower anxiety, likely due to better institutional support and preparedness (Alshammari et al., 2023).

Lastly, the finding that teaching anxiety in private high schools is lower than in ordinary high schools but higher than in key high schools remains relatively underexplored in the literature. However, existing research suggests that public school teachers generally experience lower anxiety due to greater institutional support, job security, and professional development opportunities (Agyapong et al., 2022; Khan et al., 2023). In contrast, private school teachers may face higher anxiety levels due to heightened performance pressure, parental expectations, and competitive work environments (Billote et al., 2022).

### 4.3 Measurement model

All constructs in this study were specified as reflective measurement models, as the observed indicators were assumed to be effects rather than causes of the underlying latent constructs. The table presents the indicator reliability, internal consistency reliability, convergent validity, and discriminant validity of the four main constructs: AI Literacy, Teaching Self-Efficacy, Work Engagement, and Teaching Anxiety. All constructs demonstrated strong internal consistency, with Cronbach's alpha (α) and composite reliability (CR) values exceeding the recommended threshold of 0.70 (Hair et al., 2019). Specifically, Cronbach's alpha values ranged from 0.889 to 0.936, and CR values ranged from 0.889 to 0.936, indicating high reliability. The average variance extracted (AVE) values ranged from 0.529 to 0.564, which exceed the minimum acceptable threshold of 0.50, thereby establishing convergent validity (Fornell and Larcker, 1981).

Furthermore, all item loadings were above 0.70, providing additional evidence of convergent validity. In terms of discriminant validity, the Heterotrait-Monotrait (HTMT) ratio of correlations was assessed. As shown in Table 4, all HTMT values between constructs were well below the conservative threshold of 0.85 (Henseler et al., 2015), with values ranging from 0.628 to 0.764. These findings confirm satisfactory discriminant validity among all constructs. Overall, the measurement model demonstrates satisfactory levels of reliability, convergent validity, and discriminant validity, indicating that the constructs are measured reliably and distinctly.

**Table 4:** Discriminant validity HTMT ratios.

Construct	AIL	TA	TSE	WE
AI literacy				
Teaching anxiety	0.717			
Teaching self-efficacy	0.641	0.764		
Working engagement	0.628	0.711	0.675	

### 4.4 Model fit and structural model assessment

After verifying the reliability and validity of the measurement model, the structural model was evaluated. Model fit was assessed using the standardized root mean square residual (SRMR), which was 0.076, indicating a good model fit as it is below the recommended threshold of 0.08 (Henseler et al., 2014).

In addition, *R*^2^ values represent the percentage of variance in the endogenous variables explained by the model. Hair et al. (2019) suggest that *R*^2^ values of 0.25, 0.50, and 0.75 can be viewed as weak, moderate, and substantial, respectively. Predictive relevance was assessed using the *Q*^2^ statistic, which is obtained through the blindfolding procedure. A *Q*^2^ value greater than zero indicates that the model has predictive relevance (Chin, 2010; Henseler et al., 2009). Generally, *Q*^2^ values higher than 0, 0.25, and 0.50 are interpreted as small, medium, and large predictive power, respectively (Hair et al., 2019).

**Figure 3:**
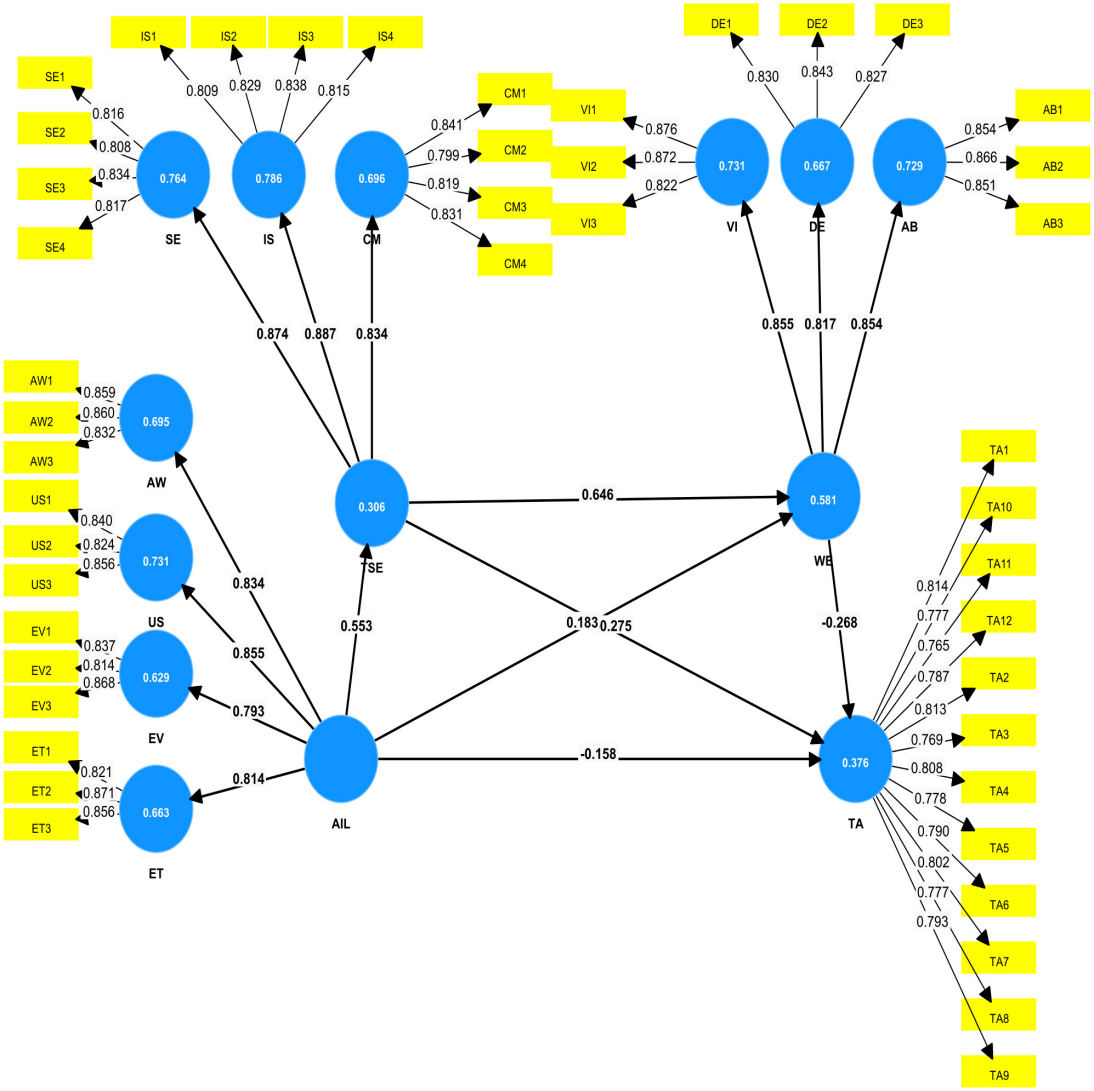
Path model.

As shown in Table 5, the R^2^ values for Teaching Self-Efficacy (TSE), Working Engagement (WE), and Teaching Anxiety (TA) were 0.349, 0.441, and 0.632, respectively. These results indicate that the model has moderate explanatory power for both TSE and WE, and substantial explanatory power for TA, which is a central construct in this study. Furthermore, the *Q*^2^ values for TSE (0.182), WE (0.244), and TA (0.353) all exceeded zero, confirming the predictive relevance of the model. Based on the thresholds outlined by Hair et al. (2019), the *Q*^2^ values suggest small predictive relevance for TSE and WE, and medium predictive relevance for TA. Lastly, the significance of the hypothesized relationships is reported in Table 6 and further illustrated in the path model diagram (Figure 3).

**Table 5:** Structural model assessment.

Constructs	*R* ^2^	*Q* ^2^	Results
TSE	0.349	0.182	Moderate/small
WE	0.441	0.244	Moderate/small
TA	0.632	0.353	Substantial/medium

**Table 6:** Results of the path analysis.

Path	Path coefficient	*t*-value	*P*-value	95% confidence interval [2.5%, 97.5%]
AIL -> TA	−0.302	7.590	0.000	[−0.380, −0.226]
AIL -> TSE -> TA	−0.227	8.557	0.000	[−0.281, −0.176]
AIL -> WE -> TA	−0.078	4.121	0.000	[−0.118, −0.045]
AIL -> TSE -> WE -> TA	−0.061	4.648	0.000	[−0.089, −0.038]
TSE -> TA	−0.384	9.412	0.000	[−0.462, −0.303]
TSE -> WE	0.423	9.606	0.000	[0.337, 0.512]
WE -> TA	−0.244	5.609	0.000	[−0.328, −0.159]

Four hypotheses were introduced by the research model for this investigation. All of the hypotheses have strong support, according to the bootstrapping analysis's results, and each were discussed in the section that follows:

Hypothesis 1: AI literacy of EFL teachers is negatively correlated with their teaching anxiety

Based on the results of the path analysis presented in Table 6, Hypothesis 1 is supported. The findings reveal a significant negative relationship between AI literacy and teaching anxiety among EFL teachers. Specifically, the path coefficient from AI literacy (AIL) to teaching anxiety (TA) was −0.302, with a *t*-value of 7.590 and a *p*-value of 0.000, indicating strong statistical significance. The 95% confidence interval for this path ranged from −0.380 to −0.226, which does not include zero, further confirming the robustness of this effect. These results suggest that higher levels of AI literacy are associated with lower levels of teaching anxiety, supporting the hypothesis that AI literacy may be negatively related to EFL teachers' teaching anxiety.

This finding is supported by Paiz (2024), who argue that teachers' AI skills are associated with lower teaching anxiety. Moreover, these findings are consistent with previous studies indicating that EFL teachers' use of AI technology is associated with higher work efficiency and lower levels of teaching pressure and anxiety (Beirat et al., 2025; Ding et al., 2024; Melweth et al., 2024; Metwally and Bin-Hady, 2025; Zhang and Zhang, 2024).

Hypothesis 2 (H2): Teaching self-efficacy mediates the relationship between AI literacy and teaching anxiety.

Based on the results shown in Table 6, Hypothesis 2 is supported. The analysis indicates that teaching self-efficacy significantly mediates the relationship between AI literacy and teaching anxiety. The specific indirect effect from AI literacy (AIL) to teaching anxiety (TA) through teaching self-efficacy (TSE) was −0.227, with a *t*-value of 8.557 and a *p*-value of 0.000. The 95% confidence interval [−0.281, −0.176] does not include zero, confirming the significance and strength of this mediation effect. These results demonstrate that AI literacy is positively associated with teaching self-efficacy, which is negatively associated with teaching anxiety, supporting the mediating role of self-efficacy in the relationship between AI literacy and teaching anxiety.

This finding is supported by Hwang and Wu (2025), who contend that teachers' AI literacy is positively associated with teaching self-efficacy and negatively associated with teaching anxiety. Furthermore, the results are consistent with previous studies suggesting that EFL teachers' integration of AI technologies is associated with higher self-efficacy and lower teaching anxiety (Deng and Liu, 2025; Fallah et al., 2023; Li, 2023; Lopez-Garrido, 2023; Zainal and Mohd Matore, 2024).

Hypothesis 3 (H3): Work engagement mediates the relationship between AI literacy and teaching anxiety.

Based on the results presented in Table 6, Hypothesis 3 is supported. The data reveal that work engagement significantly mediates the relationship between AI literacy and teaching anxiety. Specifically, the indirect path from AI literacy (AIL) to teaching anxiety (TA) through work engagement (WE) yielded a path coefficient of −0.078, a *t*-value of 4.121, and a *p*-value of 0.000. The 95% confidence interval [−0.118, −0.045] does not include zero, indicating that this mediating effect is statistically significant. These findings suggest that EFL teachers with higher AI literacy tend to report greater work engagement, which in turn is associated with lower levels of teaching anxiety.

This finding is supported by Wang and Wang (2024), who argue that improvements in AI technology are associated with teachers' use of AI technology and their participation. Moreover, these findings are consistent with previous research suggesting that higher AI literacy among EFL teachers is associated with work engagement, professional confidence, teaching efficiency, and lower levels of teaching anxiety (Chiu and Rospigliosi, 2025; Edmett et al., 2023; Fathi et al., 2024; Ramnarain et al., 2024; Sanusi et al., 2024; Zapata et al., 2024).

Hypothesis 4 (H4): Teaching self-efficacy and then work engagement serially mediate the relationship between AI literacy and teaching anxiety.

Based on the results shown in Table 6, Hypothesis 4 is supported. The data indicate that teaching self-efficacy and work engagement act as serial mediators in the relationship between AI literacy and teaching anxiety. The specific indirect effect from AI literacy (AIL) to teaching anxiety (TA) through both teaching self-efficacy (TSE) and work engagement (WE) is statistically significant, with a path coefficient of −0.061, a *t*-value of 4.648, and a *p*-value of 0.000. The 95% confidence interval [−0.089, −0.038] excludes zero, confirming the significance of this serial mediation path. This result suggests that AI literacy is positively associated with teachers' self-efficacy, which is positively associated with their work engagement and negatively associated with teaching anxiety.

This finding is supported by Hwang and Wu (2025), who argue that teachers' AI skills are associated with self-efficacy, work engagement, and teaching anxiety. Moreover, these findings are consistent with previous research suggesting that EFL teachers' AI literacy is associated with psychological needs satisfaction, teaching self-efficacy, and work engagement, which are in turn associated with lower levels of teaching anxiety (Du et al., 2024; Galindo-Domínguez et al., 2025; Shen and Cui, 2024; Xia et al., 2022; Yao and Wang, 2024; Zhang et al., 2025).

Overall, these findings suggest that AI literacy is negatively associated with teaching anxiety, and this relationship may be explained by teachers' self-efficacy and work engagement. As shown in Table 6, teaching self-efficacy emerged as a particularly strong mediator, both independently and as part of the sequential path through work engagement [β = −0.384, *t* = 9.412, *p* < 0.001, 95% CI (−0.462, −0.303); β = 0.423, *t* = 9.606, *p* < 0.001, 95% CI (0.337, 0.512)]. In addition, work engagement not only played a significant mediating role, albeit with a smaller effect size compared to teaching self-efficacy, but also showed a significant negative effect on teaching anxiety [β = −0.244, *t* = 5.609, *p* < 0.001, 95% CI (−0.328, −0.159)]. Since all confidence intervals excluded zero, both the direct effect and all three mediating pathways were statistically significant, thus providing empirical support for Hypotheses 1, 2, 3, and 4.

Lastly, Table 7 presents the mediating effects of Teaching Self-Efficacy (TSE) and Work Engagement (WE) in the relationships between AI Literacy (AIL) and Teaching Anxiety (TA). Hypothesis H2 examines the indirect effect of AIL on TA through TSE. The results show a significant direct effect of −0.384, an indirect effect of −0.227, and a total effect of −0.611. The Variance Accounted For (VAF) is 37.2%, indicating a partial mediation effect of TSE in this relationship. Hypothesis H3 investigates the mediating role of WE between AIL and TA. The direct effect is −0.244, with an indirect effect of −0.078, resulting in a total effect of −0.322. The VAF of 24.2% suggests that partial mediation is occurring, with a relatively weaker mediation effect compared to H2. Hypothesis H4 explores a serial mediation model where both TSE and WE mediate the relationship between AIL and TA. The direct effect is −0.302, the indirect effect is −0.061, and the total effect is −0.363. With a VAF of 16.8%, this result still indicates a partial mediation effect, albeit relatively weak compared to the other two hypotheses. Although the indirect effect was statistically significant, the low VAF suggests that only a small proportion of the total effect was mediated through the proposed variables (Hair et al., 2017).

**Table 7:** Mediating effect of TSE and WE.

Hypotheses	IV	MV	DV	Direct effect	Indirect effect	Total effect	VAF (%)	Results
H2	AIL	TSE	TA	−0.384	−0.227	−0.611	37.2	Partial mediation
H3	AIL	WE	TA	−0.244	−0.078	−0.322	24.2	Partial mediation
H4	AIL	TSE WE	TA	−0.302	−0.061	−0.363	16.8	Partial mediation

## 5 Discussion

### 5.1 The direct influence of AI literacy on teaching anxiety

The research results showed that EFL teachers' AI literacy had a significant negative effect on their teaching anxiety, supporting Hypothesis 1. This finding aligns with the perspectives of many scholars. For instance, Paiz (2024) emphasizes that improving teachers' AI literacy is essential for the effective integration of AI in education. Enhancing teachers' competence in using AI can help alleviate anxiety associated with implementing AI technologies in the classroom. Similarly, Beirat et al. (2025), in a study on Jordanian teachers, found that many educators encountered difficulties in applying AI tools at work, which contributed to heightened anxiety levels. They concluded that strengthening teachers' AI-related skills could significantly reduce their work-related anxiety.

Currently, the widespread integration of AI technology into EFL teaching has become a significant trend in the field of language education. As Li et al. (2024) noted, when EFL teachers observe that their peers or school leadership are actively promoting the use of AI tools, they are more likely to adopt such technologies in their own classrooms. EFL teachers with high levels of AI literacy are able to select appropriate AI tools based on specific teaching objectives and the characteristics of their students. This enables them to effectively integrate AI into various aspects of their teaching, including instruction, assessment, and classroom management, thereby enhancing both efficiency and effectiveness (Jiménez, 2024).

In contrast, EFL teachers with low AI literacy often lack the necessary skills to use AI tools proficiently. This lack of confidence and competence can create significant barriers to the adoption of AI technology in their teaching practice. Consequently, these teachers tend to experience higher levels of anxiety when faced with the challenge of integrating AI into English instruction (Melweth et al., 2024).

By enhancing their AI literacy, EFL teachers may develop a clearer understanding of both the advantages and limitations of AI tools. This understanding may be associated with lower anxiety about the possibility of being entirely replaced by AI in the teaching process (Metwally and Bin-Hady, 2025; Zhang and Zhang, 2024). Moreover, AI use may be related to teachers' completion of various instructional tasks, such as designing curriculum plans and assessing students' academic performance. When EFL teachers are proficient in using AI technologies, these tools may be linked to reduced workload and lower teaching-related stress, which in turn may be associated with lower teaching anxiety (Beirat et al., 2025; Ding et al., 2024). Therefore, improving the AI literacy of EFL teachers may be considered a factor that is associated with lower levels of teaching anxiety.

### 5.2 The mediating role of teaching self-efficacy

The teaching self-efficacy of EFL teachers plays a mediating role in the relationship between AI literacy and teaching anxiety, indicating that higher AI literacy is associated with greater teaching self-efficacy, which in turn is associated with lower teaching anxiety. This finding provides support for Hypothesis 2 and is consistent with Hwang and Wu (2025), who found that individuals' AI literacy is associated with self-efficacy and anxiety levels. According to social cognitive theory, successfully completing tasks fosters individuals' confidence in their own abilities. Repeated success, especially in overcoming challenges, strengthens self-efficacy by providing direct evidence of one's competence. Self-efficacy also plays a central role in regulating emotions, motivation, and behavior (Li, 2023; Lopez-Garrido, 2023). Muñoz et al. (2018) noted that individuals with high self-efficacy tend to experience lower levels of anxiety, worry, and social avoidance. In contrast, low self-efficacy is associated with increased anxiety and related emotional problems, such as depression and excessive worry. Individuals with high self-efficacy are more likely to perceive challenges as manageable and respond to them with confidence, and are less likely to experience anxiety. Conversely, individuals with low self-efficacy tend to perceive challenges as threats and are more likely to experience higher levels of anxiety (Deng and Liu, 2025).

When EFL teachers possess a high level of AI literacy, they may be more able to master the knowledge and skills associated with AI tools. Consequently, EFL teachers with high AI literacy may be more capable of integrating technologies such as intelligent tutoring systems and adaptive learning platforms into their teaching. These tools may enable personalized instruction, real-time feedback, and targeted interventions, which could be associated with improved teaching outcomes (Zainal and Mohd Matore, 2024). When EFL teachers experience better teaching outcomes, this may be related to increased confidence in their teaching abilities and roles, and in turn, may be associated with higher teaching self-efficacy. Higher teaching self-efficacy may be linked to lower levels of fear and pressure related to teaching challenges, which may further be associated with reduced teaching anxiety (Fallah et al., 2023). Therefore, improving the AI literacy of EFL teachers may be associated not only directly with lower teaching anxiety, but also indirectly with lower teaching anxiety through its positive association with teaching self-efficacy.

### 5.3 The mediating role of work engagement

The results of this study are consistent with H3, showing that work engagement mediates the relationship between AI literacy and teaching anxiety. Specifically, higher AI literacy is associated with greater work engagement among EFL teachers, which in turn is associated with lower teaching anxiety. Wang and Wang (2024) argued that, according to the Unified Theory of Acceptance and Use of Technology (UTAUT) framework, individual AI literacy is associated with stronger intentions to use AI, higher engagement, and lower anxiety related to technology use. Similarly, Zapata et al. (2024) conducted a statistical analysis of the literature on the impact of AI on students' mental health, finding associations between AI adoption, academic participation, stress management, anxiety levels, and well being. The results of this study are consistent with these findings, suggesting that, for EFL teachers, higher AI literacy may be associated with lower teaching anxiety through greater work engagement.

AI literacy is associated with the knowledge and skills that EFL teachers may need to effectively use AI tools. As their knowledge and skills improve, EFL teachers‘ intention to use AI in teaching tends to be higher, which may be linked to greater proactiveness in exploring new teaching methods. This, in turn, is associated with higher work engagement (Ramnarain et al., 2024; Sanusi et al., 2024). Greater dedication to work is related to a more positive attitude toward teaching, which may be associated with lower teaching anxiety (Fathi et al., 2024). Furthermore, higher AI literacy is associated with EFL teachers' ability to use AI tools to enhance their teaching performance, which may be linked to a stronger sense of professional achievement. This, in turn, is associated with increased passion for teaching and lower workplace anxiety (Edmett et al., 2023). Additionally, AI tools may be associated with improved efficiency and reduced workload for EFL teachers, which is related to better overall experience and satisfaction with teaching, and further linked to lower teaching anxiety (Chiu and Rospigliosi, 2025). Therefore, work engagement plays a mediating role in the relationship between AI literacy and teaching anxiety. Through its positive association with work engagement, AI literacy may be indirectly related to lower teaching anxiety.

### 5.4 The chain mediating role of teaching self-efficacy and work engagement

This study also found that EFL teachers' teaching self-efficacy and work engagement serve as chain mediators between AI literacy and teaching anxiety. This finding is consistent with H4, which proposes that higher AI literacy among EFL teachers is associated with greater teaching self-efficacy, which in turn is associated with higher work engagement, and ultimately linked to lower teaching anxiety. This result aligns with the perspectives of many scholars. Hwang and Wu (2025) argued that individuals' AI literacy in educational settings is positively associated with self-efficacy, which is in turn associated with higher engagement and lower anxiety. Similarly, Zhang et al. (2025) emphasized that higher AI literacy is associated with higher AI self-efficacy, which is positively associated with work engagement and negatively associated with anxiety.

From the perspective of self-determination theory, improving the AI literacy of EFL teachers can fulfill their psychological needs, thereby enhancing their teaching self-efficacy. Higher levels of teaching self-efficacy are associated with higher intrinsic motivation, which is associated with greater EFL teachers' work engagement and lower teaching anxiety (Galindo-Domínguez et al., 2025; Shen and Cui, 2024; Xia et al., 2022). As EFL teachers improve their AI literacy, they gain a deeper understanding of AI, enabling them to proficiently use AI tools to better accomplish their teaching tasks. As their teaching performance improves, so does their teaching self-efficacy (Du et al., 2024; Yao and Wang, 2024). This enhancement of self-efficacy increases teachers' sense of control and mastery over their work, further boosting their dedication, energy, and focus on teaching. When EFL teachers are confident in their teaching abilities, they may be more motivated and engaged in their work (Du et al., 2024). The increase in work engagement, in turn, is associated with lower teaching anxiety.

Although this study primarily focuses on the psychological mechanisms through which AI literacy influences teaching anxiety *via* teaching self-efficacy and work engagement, the widespread application of AI in education has also raised non-negligible ethical concerns. These ethical issues are not unrelated to teaching anxiety; rather, they may directly affect teachers' willingness to adopt AI and their emotional responses to it. Firstly, data privacy and security are among the most frequently discussed ethical concerns. AI-driven student assessment tools require the collection of large amounts of personal data (e.g., learning behaviors, performance records, and even facial expressions). If teachers are unclear about how such data are stored, used, or shared, or if they worry about data breaches or misuse, they may experience additional anxiety even when their AI literacy is high (Akgun and Greenhow, 2022). In our research model, AI literacy is associated with lower teaching anxiety through its positive association with self-efficacy; however, if teachers lack trust in the ethical safety of AI tools, this indirect association may be less pronounced.

Secondly, algorithmic bias can lead to unfair pedagogical decisions. AI systems may embed cultural, gender, or linguistic biases from training data, resulting in biased evaluations of student work or biased recommendations of learning resources. Teachers who lack the ability to identify and correct such biases may find themselves in an ethical dilemma of “whether to trust AI recommendations,” thereby triggering moral anxiety (Holmes et al., 2022). We suggest that AI literacy training should incorporate algorithmic awareness and critical evaluation skills to help teachers maintain professional judgment when faced with AI recommendations. This would be associated with higher self-efficacy and lower anxiety related to ethical uncertainty.

Thirdly, the digital divide raises issues of fairness. Significant disparities exist across schools in terms of AI infrastructure, tool quality, and training support. Teachers in under-resourced schools may not benefit from the workload-reducing advantages of effective AI, yet they are still required to use AI technologies. Such a situation of “demand without support” is associated with higher levels of work pressure and perceived inequity, and is associated with higher teaching anxiety (Lythreatis et al., 2022). We suggest that school administrators, when promoting AI integration in classrooms, should pay special attention to providing adequate training and resource support for teachers in disadvantaged schools, otherwise, the anxiety-reducing effect of improved AI literacy may be constrained by external environmental factors and fail to translate effectively into self-efficacy and work engagement.

Finally, the erosion of teachers' professional autonomy is another ethical dimension worth attention. When pedagogical decisions are increasingly guided by algorithmic recommendations, teachers may feel their own judgments are marginalized, leading to identity-related anxiety (Selwyn, 2021). If teachers perceive AI as a tool that enhances rather than replaces their professional competence (i.e., enhanced self-efficacy), their anxiety decreases. Therefore, in the design and implementation of AI tools, teachers' final decision-making power and right to override should always be preserved, positioning AI as an “assistant” rather than a “substitute.”

## 6 Implications of this study

This study has both theoretical and practical significance. Theoretically, it constructs a chain mediation model that enriches the understanding of how AI literacy is associated with teaching anxiety among EFL teachers. By revealing the mediating roles of teaching self-efficacy and work engagement, both independently and sequentially, this study advances the theoretical framework explaining the psychological mechanisms linking AI literacy to teaching-related emotional outcomes. The findings broaden the research perspective on AI use in education and offer new insights into how AI literacy may be related to lower negative emotions in teaching contexts. Furthermore, the results demonstrate that AI literacy is not only directly associated with teaching anxiety, but also indirectly associated *via* teaching self-efficacy and work engagement. This deepens our understanding of the critical role individual psychological resources play in relation to teaching anxiety, particularly in the context of rapid technological integration.

Practically, the findings provide valuable guidance for promoting AI integration in English language teaching. EFL teachers are encouraged to actively acquire AI-related knowledge and enhance their proficiency in using AI tools through online learning, peer collaboration, or formal training. These efforts may be associated with reduced teaching anxiety and improved mental well being. Meanwhile, school administrators should offer sustained support by organizing targeted AI literacy training, creating hands-on opportunities for AI application in teaching, and fostering an environment that promotes continuous professional development. Such initiatives may be linked to greater teacher confidence in using technology and lower psychological stress related to teaching in the digital era.

## 7 Limitations and future research

This study has several limitations that should be acknowledged. First, the participants were limited to high school EFL teachers from several regions in China. Given the potential influence of cultural and educational contexts on teachers' perceptions and behaviors, caution should be exercised when generalizing the findings to other countries, educational systems, subject areas, or educational levels. Second, the use of a purely quantitative design limited the ability to capture teachers' nuanced experiences and underlying mechanisms in depth. Thirdly, the study relied on self-reported data and did not include behavioral indicators of AI use in classroom practice. Therefore, the findings primarily reflect teachers' perceived experiences rather than observed behaviors. Fourth, although the BFNE was theoretically relevant to the evaluative dimension of anxiety experienced in AI-integrated teaching contexts, it was originally developed to assess fear of negative evaluation rather than domain-specific teaching anxiety. Therefore, the present study captures a specific evaluative component of teachers' anxiety rather than the full multidimensional construct of teaching anxiety. Future research could address these limitations by incorporating cross-cultural sample, mixed-methods approaches, behavioral indicators of AI use, and more domain-specific measurement instruments to further enhance validity and generalizability.

## 8 Conclusion

This study explores the chain mediating roles of teaching self-efficacy and work engagement in the relationship between EFL teachers' AI literacy and their teaching anxiety. The results show that AI literacy is not only significantly negatively associated with teaching anxiety but is also indirectly related to lower teaching anxiety through its positive associations with teaching self-efficacy and work engagement. These findings suggest that teaching self-efficacy and work engagement independently mediate the relationship between AI literacy and teaching anxiety, and together form a sequential mediating mechanism that contributes to this association. As the integration of AI technology into education continues to advance, this research offers a novel perspective on understanding lower teaching anxiety among EFL teachers. Notably, it is the first to incorporate both teaching self-efficacy and work engagement to explain the psychological mechanisms underlying this relationship. Based on these findings, it is recommended that EFL teachers actively cultivate their AI literacy, which may be associated with better management of teaching-related anxiety. Meanwhile, school administrators should provide diverse opportunities and support systems to help teachers enhance their AI competence and confidence in technology integration.

## Data Availability

The original contributions presented in the study are included in the article/supplementary material, further inquiries can be directed to the corresponding author/s.
